# Expedited batch processing and analysis of transposon insertions

**DOI:** 10.1186/1756-0500-4-482

**Published:** 2011-11-04

**Authors:** Jeremy D Smith, David A Ray

**Affiliations:** 1 Department of Biology, West Virginia University, Morgantown, WV 26506, USA; 2 Department of Biochemistry, Molecular Biology, Entomology and Plant Pathology, Mississippi State University, Mississippi State, MS 39762, USA; 3 Institute for Genomics, Biocomputing, and Biotechnology, Mississippi State University, Mississippi State, MS 39762, USA

## Abstract

**Background:**

With advances in sequencing technology, greater and greater amounts of eukaryotic genome data are becoming available. Often, large portions of these genomes consist of transposable elements, frequently accounting for 50% or more in vertebrates. Each transposable element family may have thousands or tens of thousands of individual copies within a given genome, and therefore it can take an exorbitant amount of time and effort to process data in a meaningful fashion.

**Findings:**

In order to combat this problem, we developed a set of bioinformatics techniques and programs to streamline the analysis. This includes a unique Perl script which automates the process of taking BLAST, Repeatmasker and similar data to extract and manipulate the hit sequences from the genome. This script, called Process_hits uses an object-oriented methodology to compile all hit locations from a given file for processing, organize this data into useable categories, and output it in multiple formats.

**Conclusions:**

The program proved capable of handling large amounts of transposon data in an efficient fashion. It is equipped with a number of useful sub-functions, each of which is contained within its own sub-module to allow for greater expandability and as a foundation for future program design.

## Findings

Modern sequencing methods have resulted in a tremendous increase in the amount of genomic sequence data available. For example, as of this paper, the NCBI Entrez Genome Project database contains ~400 eukaryotic genomes that have been completed or are in the process of being assembled [1]. Most of these genomes consist of large transposable element (TE) derived fractions. For example, transposable elements account for 45% of the human genome, 37% of the mouse genome, 73% of the maize genome, and 26.5% of the zebrafish genome [2-5]. TEs are valuable tools for genetic analysis and modification. They also represent drivers of genomic evolution and diversification by acting as mutagens or as targets for non-homologous recombination. In addition, TEs can act as sources of novel protein coding sequences and regulatory motifs [6,7].

As a result of their prevalence and importance in genome biology, TEs are important study subjects. However because of their repetitive nature, specific techniques are required to deal with them. For example, researchers may be interested in investigating patterns of evolution among TE families or the distribution of the elements in the genomic environment. In cases where the genome is relatively unexplored, this can be difficult. The initial step is often identifying the transposable element families present in a given genome. This can be done *de novo *using tools such as RepeatScout [8], PILER [9], or a variety of other tools [10], discovering and detecting transposable elements in genome sequences [11], by comparison to a related organism, or by obtaining a previously generated library, such as those provided by RepBase [12].

Once a basic TE library is developed, it can be used to search for specific insertion sites within a genome. BLAST [13] and RepeatMasker [14] are two tools that are commonly used. RepeatMasker identifies and masks transposable elements using a known library, while BLAST will output matches to query sequences based on the quality of the match. However, unlike when dealing with a single or small set of genes, transposable element searches using either tool can return anywhere from a few hundred to millions of hits for each element family present within a given genome. Previously, there have been few good options for dealing with this amount of data. While there are several options for converting BLAST to FASTA, most of them seem to only work for a single sequence at a time or do not work with local BLAST data and while it is possible to manually extract the appropriate sequence information from BLAST or RepeatMasker output, doing so can be time consuming.

In order to counter this problem, we developed a set of bioinformatics techniques and programs to streamline analyses. These tools include a unique set of Perl scripts which automate the process of taking BLAST, Repeatmasker or similar data, locating the hit sequence, and exporting these sequences with unique IDs to a new FASTA file. This tool, called Process_hits, uses a combination of object-oriented and BioPerl [15] methods to compile all hit locations from a given output file for processing, organizes this data into useable categories, and outputs it into a variety of formats, allowing for the processing of large amount of sequence data over a short period of time.

### Functionality

Process_hits was designed with each of the common major sub-functions (hit processing, sequence extraction, and hit-object methods) contained within their own sub-modules. This program is composed of process_hits.pl, Geneloc.pm, Gene_process.pm, and Gene_extract.pm as well as several optional simple scripts designed to assist with pre-formatting and other data management issues. Process_hits.pl is the user interface; it takes in data and provides a framework for calling the library modules. Gene_loc.pm contains the modules for creating Geneloc objects, which store information on sequence location within a FASTA file. Gene_process.pm contains the modules required to convert input data into Geneloc objects, while Gene_extract.pm contains the modules required to convert Geneloc objects into FASTA sequences and output the data in several different methods. The optional scripts include Clean_FASTA, Split_BLAST, and Split_Repeatmasker. Clean_FASTA removes any characters or unusual formatting that may be present in the nucleotide sequences of a FASTA file. Split_BLAST and Split_Repeatmasker will break a single file down into smaller subfiles based on a given parameter, similar to the functionality provided by Process_hits described below.

User input is limited to process_hits.pl and consists of RepeatMasker formatted, EPCR tabular formatted [16], or tabular BLAST output formatted text files and a FASTA formatted sequence file. Variable parameters are set as part of the command line input. Usage options include a log function and a verbose function to assist with testing. Input options include using a single file or a list of files. The implemented quality control parameters allow users to set the minimum and/or maximum length, the maximum number of gaps, the maximum number of mismatches, and in the case of BLAST files, the minimum bit score or E-value for a given sequence. For BLAST data, the user can choose to output only a given number of the best hits based on the E-value. One of the advantages of these optional parameters is that one can perform a single BLAST or RepeatMasker search and use Process_hits to select only a specific portion of one's data for analysis.

Geneloc.pm provides the base methods that are necessary to create Geneloc objects. Each Geneloc object is established with a default set of attributes, which may be expanded automatically without specifying a new method. The default attributes include hit_ID, location, seqStart, seqEnd, and seqLength. Hit_ID contains the unique name for each object, and location contains the name of the sequence where the sub-sequence described was found. Both seqStart and seqEnd are numerical values for the site of the sub-sequence within a greater sequence, while seqLength is defined as the distance between the two values.

The input file is opened in Gene_process.pm and converted into Geneloc objects line by line. Geneloc objects are stored within a single array. This conversion process corrects for sequence orientation where it is needed. Once the entire file has been processed, the Geneloc objects that contain overlapping or adjacent sequences, may be combined into a single Geneloc object as set by the user. These Geneloc objects are then checked against the given parameters and removed if they do not fall within the given quality controls. Here, the data may be split into subgroups based on the query sequence for BLAST or the element name or family for RepeatMasker. For every unique query or element a new array containing the matching Geneloc objects is created. These new arrays are then contained within a hash with unique keys based on the individual query or element.

The array or arrays containing the Geneloc objects are then sent to Gene_extract.pm for extraction. The default output option is a single unaligned FASTA file containing all the matching sequences within the range of the parameters given above, but if one of the split methods detailed above is used, multiple FASTA files will be created. If this option is used, the program can also retrieve the original query sequences from a given FASTA file and match those sequences with the Geneloc objects. Initial FASTA files are indexed using the BioPerl method Bio::DB::Fasta [9]. Geneloc objects are extracted one by one using their Start, End, and Location methods. The Start and End methods can be modified by adding a buffer that expands the sequence beyond the parameters of the original hit. This will extend the extracted sequence upstream or downstream where data is available. Using the indexed database, each Geneloc object is retrieved and printed to the appropriate FASTA file. If the output consists of similar sequences, there is an option to align all sequences, align the subsets created using the split methods, or align a random selection of them using MUSCLE [17], which if combined with the query retrieval methods above, will align the output sequences with their original query sequence. Other output options include a text file in the appropriate format of the final Geneloc objects to be extracted, which can be combined with a no-extract option in order to output only the text file.

### Demonstration

**Figure 1:**
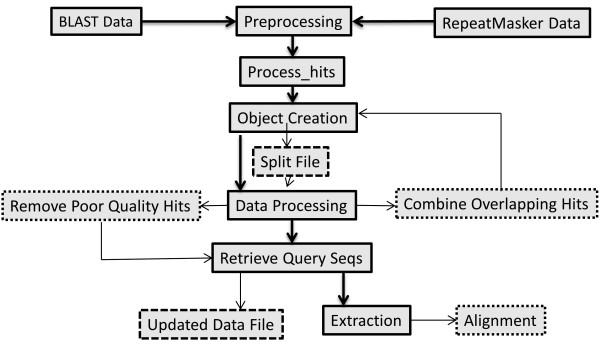
**Process_hits workflow**. The workflow of the Process_hits package is pictured here. Heavy arrows and solid boxes are the default methods. Light arrows and dashed boxes are optional methods.

Here we give a step-by-step demonstration of how to use Process_hits and examples of the most commonly used options (Figure 1). The input files required are a FASTA-formatted sequence database and a "hit" file from one of several search programs. The FASTA-formatted sequence database file will be the file used in the original BLAST or RepeatMasker search. The "hit" file is either a tabular output file created by BLAST or the standard. out file created by RepeatMasker. For the purposes of these examples, we will use ExampleDB.fas as the database file, BLASTExample1.out as the BLAST output file, and RMExample1.out as the RepeatMasker output file. All three files can be downloaded from the Examples folder on the Process_hits Sourceforge page.

For the first example we will use a BLAST output file as part of the initial search for the transposable element content of a given sequence database. To begin, the user opens a command window or terminal and directs it to the working directory. The Process_hits scripts will need to be placed in either this directory or in an executable folder (*bin on most Linux systems). Next comes the following command: "process_hits.pl -input blast -v -buffer 200 -bit 100 -minlength 100 -splitquery -align -print BLASTExample1.print.txt BLASTExample1.out ExampleDB.fas". The 'v' parameter will set Process_hits to verbose mode so that the user can follow along as the program runs and check for any potential errors running it for the first time on a new machine or dataset. Similarly, 'print' will create a tabular format BLAST file with only the BLAST hits that were extracted, which can be useful for error checking and so that the user will know exactly what is going into and coming out of the program. 'Minlength' and 'bit' are quality control options, extracting only sequences of a certain length and of a given bit score. The remaining three parameters control the Process_hits output. 'Buffer' will extract 200 bases upstream and downstream of the original hit and is typically used to either obtain flanking sequence or for expanding outward in order to retrieve a full-length transposable element sequence from your database. 'Splitquery' and 'align' will split the FASTA output into a separate file for each matching query sequence and align the output files using MUSCLE. Taken together, these options make a solid approach to an initial BLAST search for individual or multiple transposable element families.

For a RepeatMasker example, one typical application is to search for the 5' and 3' ends of a transposable element family. The method is designed to extend the original query and eventually find examples of full length sequences of larger element, such as LINEs. Again, the user starts by opening a command prompt or terminal into the directory containing the user's working files as well as Process_hits. The user proceeds by sending the following command: "process_hits.pl -input repeatmasker -splittype -align -overlap 100 -buffer 1000 -minlength 500 -qseq ExampleQuery.fas RMExample.out ExampleDB.fas". Here larger 'buffer' and 'minlength' options are used so that longer extracts are retrieved in an attempt to identify the full longer hits to the TEs of interest. Since in this case longer extracts are being retrieved, we have added the 'overlap' parameter, which will combine any hits within 100 bases of each other before buffering. This allows nearby hits that are likely to be from the original insertion but separated by small indels to be combined into the same extracted sequence. 'Splittype' functions are similar to 'splitquery' as described above. 'Qseq' will retrieve the original query sequences from a given FASTA file and add them to the FASTA files that are generated prior to alignment ("-align"). When taken all together, these settings will allow you to extend the partial TE sequences that are often output by PILER, RepeatScout, RepeatModeler, and other *de novo *repeat identifiers and generate a full length consensus sequence. It is also useful for the analysis of the flanking sequence of selected TEs.

Additional examples and details on various parameters and options can be found in Examples.txt and the readme.txt files, which are part of the Process_hits distribution.

## Conclusions

Process_hits is able to handle large amounts of TE data in an efficient fashion. The Process_hits package has proven capable of extracting 68,820 sequences in 68 seconds as detailed in Table 1. The indexing methods described above are significantly faster when dealing with greater than a hundred hits than either BLAST-search or memory-intensive methods that had been previously used. However, use of multiple quality selection parameters, the overlap function, or splitting a large file can slow the process considerably. The combination of modular design and object-oriented processes allow for easy implementation of new formats and customizable outputs, as well as providing the foundation for future program design. The Process_hits program and related scripts have been uploaded to Sourceforge.net where they should remain available perpetually and continue to receive regular updates.

**Table 1:** Benchmarks

	Size (Mb)	Sequence Number	Hits Extracted	Extraction Time (s)
*Anolis carolinensis *(AnoCar2.0)	1817	7233	68820	68

*Arabidopsis thaliana (*TAIR9.171)	97	1447	9506	4

*Aspergillus nidulan *(FGSC A4 s06-m03-r02)	31	248	459	1

*Homo sapiens* (GRCh37)	3147	47650	254941	118

*Loxodonta africana *(loxAfr3)	3785	233134	52874	135

*Macropus eugenii *(Meug_1.1)	3013	616418	24627	145

*Oryctolagus cuniculus *(OryCun2.0)	3537	215471	53097	121

*Petromyzon marinus *(petMar1)	1049	108241	11086	35

*Zea mays* (B73 RefGen v2)	2095	31719	246699	100

All benchmarks were performed on a Dell PrecisionT3500 with Intel Xeon E5530 2.40 GHz processor and 6 GB of RAM running Windows XP 64. Example organisms are listed in the first column with the genome version listed in parenthesis. The Size column lists the genome database used in megabytes. The Sequence Number column lists the number of FASTA sequences found in the database file. The Hits Extracted column lists the total number of BLAST hits extracted from the database file. The Extraction Time lists the time in seconds required to extract the Hit Objects from the FASTA-formatted genome database.

## Availability and requirements

**Project name**: Process_hits

**Project home page**: https://sourceforge.net/projects/processhits/

**Operating systems**: Platform independent

**Programming language**: Perl 5.10.0+

**Other requirements**: BioPerl 1.61+

**License**: Academic Free License (AFL)

## Competing interests

The authors declare that they have no competing interests.

## Authors' contributions

The package was originally conceived by JDS and DAR, based on the research of DAR. JDS designed and wrote the package. DAR assisted with testing the software as part of ongoing genomic analyses. JDS wrote the draft of the article, which was revised by DAR.
